# Intelligent Technical Textiles Based on Fiber Bragg Gratings for Strain Monitoring †

**DOI:** 10.3390/s20102951

**Published:** 2020-05-22

**Authors:** Petr Munster, Tomas Horvath

**Affiliations:** Department of Telecommunication, Brno University of Technology, Technicka 12, 616 00 Brno, Czech Republic; horvath@feec.vutbr.cz

**Keywords:** intelligent textile, FBG, strain, temperature

## Abstract

In this paper, the concept design of intelligent technical textile blocks implemented with optical fibers that include fiber Bragg gratings for strain and temperature sensing is briefly introduced. In addition to the main design of the system, a design of measurement blocks with integrated fiber Bragg grating elements for strain measurement is also presented. In the basic measurement, the created textile block was tested for deformation sensitivity when a load was applied. Moreover, a unique robust and low profile connector was designed, created and verified. The fibers are terminated with GRIN lenses, allowing easy manipulation and completion of the connector in the field, with an average insertion loss of 5.5 dB.

## 1. Introduction

Intelligent technical textiles are functional industrial textiles implemented with optical sensor elements for distributed and quasi-distributed sensing of temperature, pressure, deformations, and chemical changes (leakage of liquids, vapor, etc.) for monitoring of critical infrastructures or their surrounding environment.

The main application of intelligent textiles is the detection of unwanted and serious events associated with human intervention, natural disasters or technical failures.

Several papers describe fiber Bragg grating (FBG)-based smart textiles for various types of applications. For example, the authors in [1] describe FBG-based smart textiles for monitoring respiratory movements for healthcare applications. The authors in [2,3,4] also focus on healthcare. For example, paper [5] presents an experimental assessment of a male-fit smart textile based on twelve FBG sensors for monitoring of respiratory parameters. Another application is intelligent building construction, which is presented by Bremer et al. in [6]. Other articles are dedicated to the usability of textiles and smart clothing [7,8,9,10,11,12].

In [13], the authors describe various types of energy-harvesting textiles that can detect changes in their surroundings and react to them based on piezo-, pyro-, ferro- and dielectric materials. The authors in [14] describe smart textiles for monitoring composites with flexible adapted sensors and actuators that detect stress, deformation, temperature changes, light intensity, and other signals from the environment. The presented smart textile is based on microelectronic sensors, whose main disadvantage is sensitivity to electromagnetic fields.

In [15], the authors precisely describe wearable fiber optic technology based on smart textiles for fashion and aesthetic purposes, disease treatment, and healthcare monitoring.

In [16], smart technical textiles based on several fiber optic sensors are described. The author compares the Brillouin optical time-domain analysis (BOTDA) sensor with FBG-based sensors and low-priced standard plastic optical fiber (POF), implemented in geotextiles for structural health monitoring, using an optical time-domain reflectometer (OTDR).

The authors in [17] describe smart textiles based on fiber optic technology for medical applications. They use textile-based fiber optic sensors in a magnetic resonance imaging environment, where standard electronic sensors cannot be employed.

The motivation of the paper is to verify the design of an intelligent textile block with FBG elements and a new type of fiber optic connector for connecting multiple blocks together. The advantage of the proposed solution lies its simplicity, cost and possibility of integration with optical data transmission systems. Optical fiber infrastructure is crucial for all types of data transmission and should be protected. In addition to protection of the optical infrastructure itself, other events can be monitored in parallel, and such a system will contribute to the protection of other critical infrastructures, such as railways and bridges. The deployment of distributed sensors along existing telecommunications routes will create a smart infrastructure.

The main contribution of this paper is the presentation of a concept design for temperature and strain monitoring by FBG sensing elements implemented in nonwoven textiles and verification measurement of strain applied to the textiles. A unique system of measurement blocks connected by a special thin connector based on GRIN lenses was proposed. Due to the small dimensions, the blocks can be connected and the connector can be placed inside the textiles. The composition of the materials and small dimensions of the measurement elements ensure the optimal distribution of the measured strain and temperature. Owing to this capability, it is possible to localize mechanical stress and temperature caused by ambient influences. This modular system can be used for several applications such as monitoring critical infrastructures (dams, tunnels, bridges, etc.) and monitoring the movement of undesirable persons in restricted areas (airports, borders, and dangerous places). The system can also be used in industrial applications for container or pipe monitoring.

The rest of this paper is structured as follows. Section 2 addresses the methodology and provides a theoretical description of the system. In Section 3, verification of the designed textile block is presented. Section 4 demonstrates the proposed home-developed special connector based on GRIN-lens technology. Finally, Section 5 concludes the paper.

## 2. Methodology and Overall System Concept

**Figure 1:**
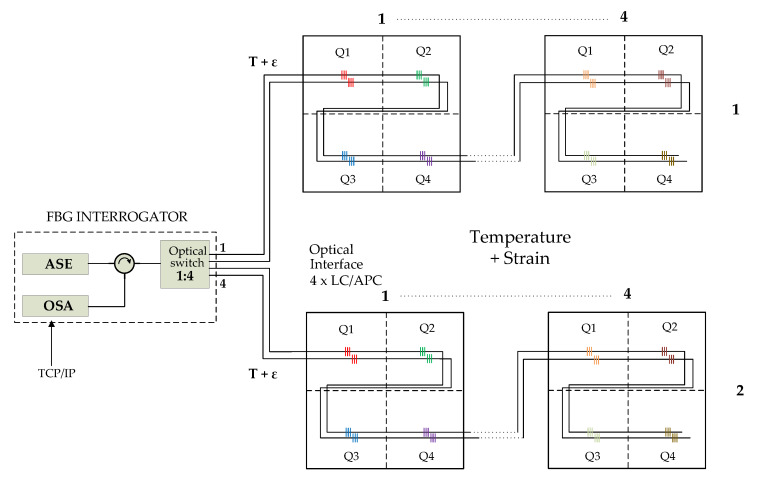
Whole concept of the system.

The concept of the developed system is based on two basic elements—the evaluation (interrogation) unit and partial blocks with dimensions of 1 m × 1 m forming the final measurement area. The basic idea was presented in [18]. Each block consists of 4 quadrants, as shown in Figure 1. The partial quadrants contain one measurement element in the case of temperature measurement only or two elements in the case of simultaneous temperature and thrust (mechanical deformation) measurement. The evaluation unit has a 4-physical-channel Lucent connector/angled physical contact (LC/APC) connector, with up to 16 sensor elements per grid, in the case of our FBG grid. The key element here is the optimally spaced spectrum and spectral spacing between the individual sensors so that when their wavelength changes due to variations in the measured quantity, there is no mutual spectral overlap with neighboring sensors. The overall spectral range of the evaluation unit is 40 nm for each physical channel within the C-band range. Table 1 provides a comparison of commercially available units with the units used for measurement and evaluation of the results. As seen from Table 1 the proposed system has parameters comparable to or better than those of commercially available systems. However, the proposed system has no defined dimensions because it will be available in the market in the second half of 2020, which is why these values are not specified. Additionally, Table 1 does not contain the price because it is defined customer by customer according to their requirements.

### 2.1. Temperature Measurement

When measuring only the temperature, it is possible to connect 4 textile blocks to each physical channel. The total measurement system capacity is then 16 textile blocks (4 channels of 4 blocks), i.e., 64 quadrants. With the proper distribution of individual channels and sensors in the spectrum, it is possible to measure the temperature in the range of −30 °C to +180 °C in each quadrant. The channel spacing for the measurement range of 220 °C at the FBG grid sensitivity of 10pm/°C is 2.2 nm.

### 2.2. Strain Measurement

When measuring the tensile strength (mechanical deformation), it is necessary to compensate for temperature changes; i.e., in addition to the thrust itself, the temperature must be measured by an independent sensor so that it is possible to compensate for the wavelength changes caused by changes in the ambient temperature to avoid interference. It follows that the total measurement capacity must be reduced by half the number of measurement quadrants since it is necessary to use 2 physical channels to connect 4 textile blocks, i.e., only two textile blocks per channel. Thus, the total capacity decreases to 8 textile blocks, i.e., 32 quadrants. The principle of the thrust measurement is shown in Figure 1. With the appropriate distribution of the individual channels in the spectrum, it is possible to measure the temperature in the range of −30 °C to +180 °C and a strain of 1500 μϵ in each quadrant. The channel spacing is the same as that in the previous case (2.2 nm), which corresponds to measurement ranges of 220 °C and 1500 μϵ at FBG sensitivities of 10 pm/°C and 1.3 pm/μϵ. The spectral distribution for the individual sensors is the same as for the temperature measurement.

### 2.3. Design of Sensor Elements

**Figure 2:**
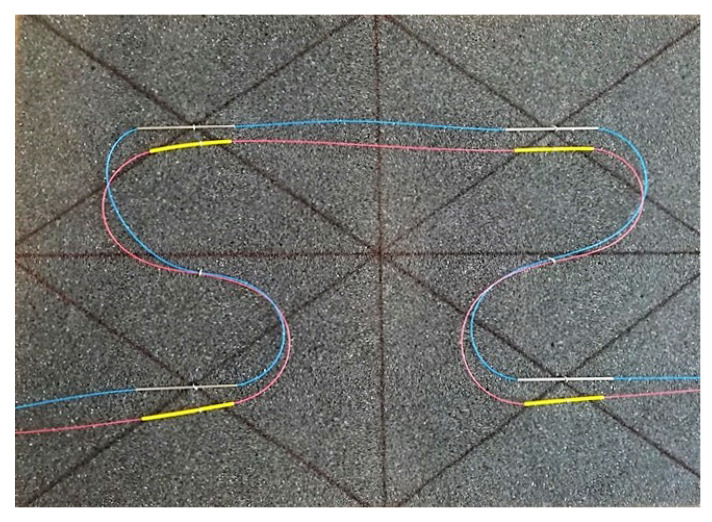
Model of the meander for strain and temperature measurement.

Figure 2 shows a model of the measurement block at a scale of approximately 1:10. This illustrates the possible arrangement of optical fiber meanders implemented in industrial textiles. Optical fiber (with appropriate secondary protection) is routed through the textile blocks to measure the temperature, with the FBG grid written at its own measurement point on a bare fiber protected by a 0.8 mm thin stainless steel capillary. The bare fiber is within a stainless steel capillary that is placed loosely and is fixed at the ends of the capillary with an optimum epoxy adhesive for bonding the optical fiber to stainless steel.

To measure the strain, it is necessary to implement two optical fibers in the textile block, as shown in Figure 2, or two FBG grids can be written to measure the temperature and strain and can be placed in close proximity. For the design of the strain sensor, acrylate recoating (recovery of primary protection) of the written FBG is applied.

As a result of stretching, the wavelength changes, but at the same time, the wavelength also changes due to changes in the ambient temperature (the FBG sensitivity is very high at approximately 10 pm/°C); therefore, slight changes due to the temperature significantly affect the measurement accuracy and must be compensated. To compensate for temperature changes, it is advantageous to use both measurements simultaneously.

## 3. Verification of the Functionality of the Designed Textile Block for Strain Measurement

In the first phase, only FBG sensing elements for strain measurement were implemented. Based on the measurement as a suitable basis, a polyester fabric with a grammage of approximately 300–400 g·m^−2^ was chosen because of its better tensile strength and elasticity than those of polypropylene fabric. RONAN FIX with a metric weight of 160 g·m^−2^ was used for reinforcement.

**Figure 3:**
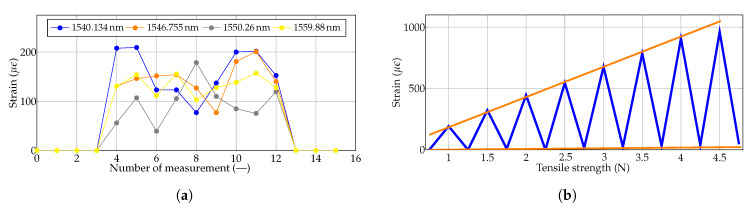
Strain changes caused by (**a**) a 6 kg load and (**b**) the tensile stress caused by a force gauge.

Experimental testing with a load of 6 kg was performed to verify the behavior of all 4 strain sensor elements implemented in textiles under load. Figure 3a) shows the graph for when a 6 kg-weight load was placed randomly around the FBG elements. As seen from the graph, the strain changes caused by the load are not constant. The most important parts are at the beginning and the end of the measurement, where it can be seen that the fabric returns to its original state when no load is applied. Figure 3b) shows a precise measurement of the strain caused by uneven tensile action on the textile block. There is a linear dependence of the strain on the tensile stress and a slight deformation of the fabric when relieving the tension. This deformation causes a measurement error of approximately 3 %. As seen in Figure 3 until 4 N, the relation is almost linear. However, for higher forces, the dependence is not linear. The aim of the measurement was to verify the functionality of the textile block. The measurement error was determined based on the deformation of the textile block when relieving the tension. In other words, when a 4.5 N force was applied, the reference value (zero point) changed by approximately 3 %. In these measurements, temperature elements were not implemented in the fabric, but to eliminate temperature error, another FBG that was not affected by strain was used for temperature measurement.

## 4. Connection of Measurement Blocks

**Figure 4:**
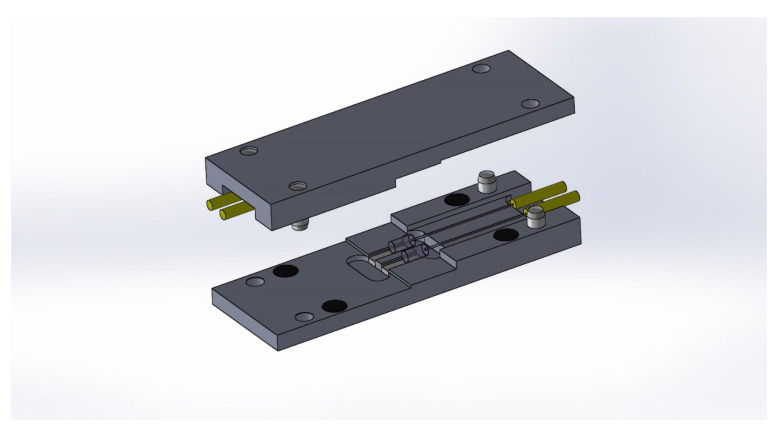
Design of the connector for an intelligent technical textile block.

Because technical textile blocks are often considered in series, a special connector based on GRIN-lens technology is proposed. In the first step, several potential connectors were designed in SolidWorks (see Figure 4, and the best solution was chosen for our connector.

**Figure 5:**
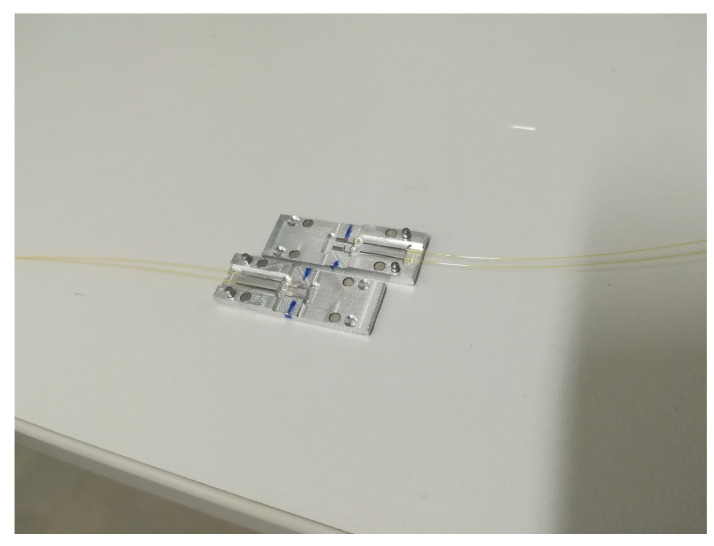
Semifinished connector product.

The principal of this connection is accurate guidance of optical fibers and especially GRIN lenses, which are spliced to standard telecommunication fiber G.657.A2. Precise V-grooves ensure alignment of cylindrical lenses with each other. In this type of connection, it is necessary to eliminate axial, angular and transverse deviation, among which angular deviation is the major cause of attenuation. GRIN lenses were spliced by a special 3 electrode splicing system with ring of fire technology, and V-groves were made with a femtosecond laser. An important part of the design is that lenses are aligned in long grooves on both sides—i.e., the design is composed of two symmetrical parts. The connector was designed for two fibers—strain and temperature (compensation) sensory elements. Based on a model created in SolidWorks, a real semifinished product was made by a computer numerical control (CNC) milling machine (see Figure 5) [24]. After CNC milling, the V-grooves were subjected to a femtosecond laser.

**Figure 6:**
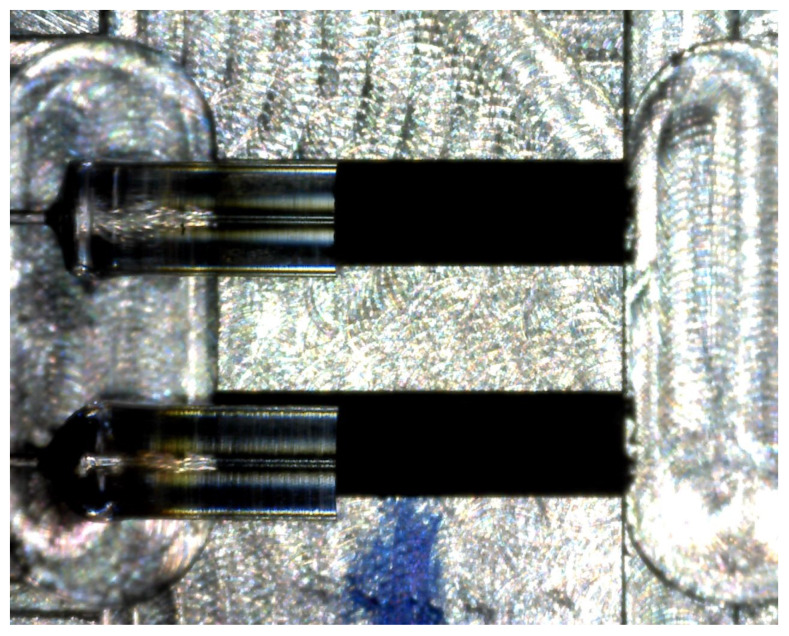
Detail of lenses in the connector.

Neodymium magnets were used for detachable fixation of the two coupling halves, and metal pins were used for precise alignment of both parts of the connector. Optical fibers were glued to the primary polyimide coating to prepare V-grooves. GRIN lenses were loosely inserted into the V-grooves (Figure 6) and aligned after both parts were fixed.

The V-grooves force the lenses into a defined position, which ensures minimal axial, angular and transverse deviation.

An undisputed advantage of the designed connector is the size of only 30 × 10 × 4 mm because the connector must be inserted in a join between two measurement blocks. Standard telecommunication connectors cannot be used for this purpose due to their large dimensions. Another advantage is the easy assembly and disassembly of the connector while maintaining high repeatability and low attenuation. Table 2 shows the attenuation of both lens-to-lens connections for 10 reconnections. The measured attenuation is between 5 and 6 dB, with very good repeatability. Although the average attenuation is approximately ten times that for standard ferule connectors, it is sufficient because the dynamic range of most FBG interrogators is approximately 30–40 dB, so this connector type can be used for connection of up to 6 measurement blocks.

**Figure 7:**
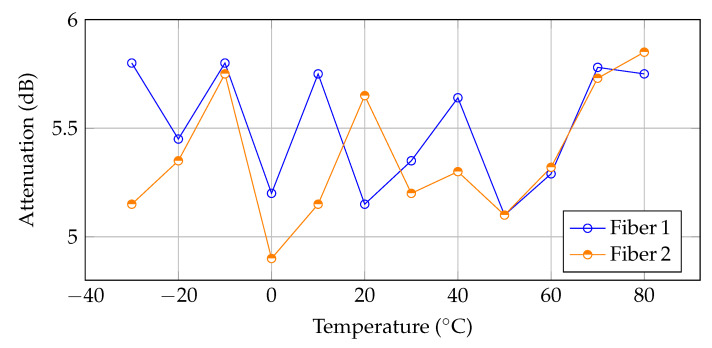
Influence of the temperature on the attenuation of the connector.

An important parameter for use in real ambient conditions is the attenuation dependency on the temperature. Continual attenuation measurements were performed in a climatic chamber in the temperature range of −30 °C to 80 °C. As seen from Figure 7 the attenuation was still in the range of 5–6 dB. This result leads to the possibility of using the connector in real conditions, and it can be used as a connector for intelligent technical textiles.

## 5. Conclusions

In this paper, the usage of technical textiles as intelligent technical textiles for strain and temperature monitoring was briefly introduced. The main application of smart textiles should be to protect critical infrastructures and detect leakage of liquids or movement of persons in restricted areas. A special connector was created for connecting individual textile blocks, which is thermally stable, achieves an insertion loss below 6 dB and is mechanically resistant. Owing to the high resolution of the used interrogator (Network Group Complex with a resolution of 1 pm), it is possible to precisely evaluate not only the measured parameters but also the measurement error caused by changes in the fabric dimensions caused by high tension.

The direction of future work will be optimization of technical textiles and design of graphical user interfaces (GUIs) to obtain a better representation of the results.

## Figures and Tables

**Table 1:** Comparison of commercially available units with the home-developed system [19,20,21,22,23].

Producer	Type	Measurement Range [nm]	Scanning Frequency [Hz]	Number of Channels	Sensors per Channel	Resolution [pm]	Dimension (W×D×H) [mm]
Welltech	FBG-S-121-32	41	1	4–32	18	1	360×300×100
Optics11 FAZ Technology	FAZT I4-16	39.2	1	up to 30	16	1	324×276×116
Ausoptics	FI3100	35	10	1–2	12	1	170×280×95
Sylex	SCN-44 S-line Scan 404	40	0.5	4	NA	1	300×200×84
Network Group	Static	7	0.3	4	4	0.1	230×115×80
Network Group	Complex	80	10	1–32	20	1	TBA

**Table 2:** Attenuation of the proposed connector.

Connection No.	Fiber 1 IL (dB)	Fiber 2 IL (dB)
1	5.3	4.9
2	5.7	5.1
3	5.1	5.6
4	5.4	5.2
5	5.6	5.3
6	5.1	5.1
7	5.2	5.6
8	5.7	5.7
9	5.6	5.2
10	5.3	5.3

## Abbreviations

The following abbreviations are used in this manuscript:

APC	Angled physical contact
ASE	Amplified spontaneous emission
BOTDA	Brillouin optical time-domain analysis
CNC	Computer numerical control
FBG	Fiber Bragg grating
GUI	Graphical user interface
IL	Insertion loss
LC	Lucent connector
OSA	Optical spectrum analyzer
OTDR	Optical time-domain reflectometer
POF	Plastic optical fiber
